# Get4/5-mediated remodeling of Get3's substrate-binding chamber: Insights into tail-anchored protein targeting by the GET pathway

**DOI:** 10.1016/j.jbc.2025.110667

**Published:** 2025-09-01

**Authors:** Diego Granados-Villanueva, Andrew Rossow, Kelly H. Kim

**Affiliations:** 1 Department of Biochemistry and Molecular Biology, Michigan State University, East Lansing, Michigan, USA; 2 Department of Biochemistry, University of Wisconsin-Madison, Madison, Wisconsin, USA

**Keywords:** protein targeting, cryo-EM, protein complex, protein–protein interaction, GET pathway, tail-anchored protein, chaperone, membrane protein

## Abstract

The Guided Entry of Tail-Anchored Proteins (GET) pathway ensures accurate targeting of Tail-Anchored proteins (TAs)—a diverse class of membrane proteins—to the endoplasmic reticulum (ER) membrane. In yeast, newly synthesized TAs are captured by Sgt2 and transferred to Get3 for delivery to the ER, where they undergo subsequent membrane insertion. Efficient and protected handoff of hydrophobic TAs from Sgt2 to Get3 is facilitated by the Get4/5 complex, which is thought to act as a scaffold to position TA-bound Sgt2 and Get3 in proximity while trapping Get3 in an ATP-bound conformation necessary for TA binding. To define the molecular basis for this process, we determined the cryo-EM structure of the *Saccharomyces cerevisiae* Get3-Get4/5 complex at 3.2 Å resolution. Our structure shows that Get4/5 remodels Get3's TA-binding chamber by unfolding helices that form the lateral walls of the chamber. We termed this region the “lateral gate,” as its helix-to-coil transition makes the TA-binding chamber more solvent accessible. Molecular dynamics simulations highlighted the flexibility of the lateral gate, indicating it is structurally dynamic and prone to conformational changes. Mutagenesis studies showed that the lateral gate residues influence both the binding affinity of Get3 for Get4/5 and its ATPase activity. Additionally, our cryo-EM map shows that the Sgt2-binding domain of Get5 is positioned near the lateral gate opening of Get3's TA-binding chamber. Based on these findings, we propose a model in which Get4/5 opens Get3’s TA-binding chamber to form a lateral opening, enabling protected, lateral transfer of TAs from Sgt2 to Get3.

Membrane proteins are essential for various cellular processes, such as signal transduction, transport, and energy production (1), and their accurate localization to specific membrane compartments is vital for optimal function (2). Many membrane proteins are initially targeted to the endoplasmic reticulum (ER) before being directed to their final destinations (3). While many of these proteins follow the co-translational pathway, relying on the signal recognition particle (SRP) to direct nascent polypeptides to the ER membrane as they are being synthesized, others are targeted post-translationally (4). Unlike co-translational targeting, post-translational membrane protein targeting faces the unique challenge of capturing and delivering fully synthesized membrane proteins to the ER while preventing their aggregation in the aqueous cytosol (5).

The yeast Guided Entry of Tail-anchored proteins (GET) pathway serves as a model system for studying post-translational membrane protein targeting mechanisms. This pathway specifically targets and inserts tail-anchored (TA) proteins, which are characterized by a single transmembrane domain at their C-terminus (*i.e.*, the “tail”), to the ER membrane (6). TAs are functionally diverse and play crucial roles in processes such as vesicular trafficking and protein translocation. Examples include SNARE proteins, such as Sed5, involved in vesicular trafficking, as well as components of the protein translocation machinery, like Sec61β (7, 8, 9). Accurate ER targeting of these newly synthesized TAs by the GET pathway is essential for maintaining these critical cellular processes (9). When the GET pathway is disrupted, TAs fail to insert into the ER membrane properly and instead accumulate or aggregate in the cytosol (10, 11). In mammals, loss of the homologous TRC pathway results in severe developmental defects, including embryonic lethality (12).

The GET pathway is a highly conserved, ATP-dependent pathway that relies on a series of coordinated steps carried out by dedicated protein components as follows (8) (Fig. 1*A*). Initially, the cytosolic chaperone Sgt2, the most upstream component of the GET pathway, binds the hydrophobic C-terminal tails of a newly synthesized TA to prevent their aggregation in the cytosol (13). The TA is then transferred to the central targeting factor Get3, an ATPase that functions as a molecular shuttle to deliver the TA to the ER membrane (14). At the ER membrane, the Get1/2 insertase complex receives the TA from Get3 and catalyzes its insertion into the membrane (10).

**Figure 1:**
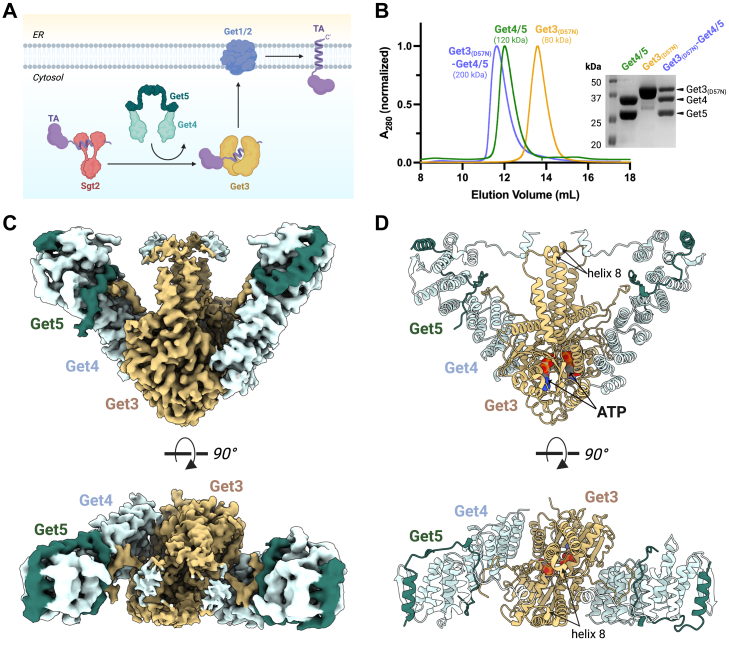
**Cryo-EM structure of the yeast Get3_(D57N)_-Get4/5 complex.***A*, schematic diagram illustrating the current model for TA targeting to the ER by the yeast GET pathway. A newly synthesized TA is first captured by Sgt2 and then transferred to Get3 in a process that is mediated by Get4/5. The ER membrane insertion of the TA is facilitated by the Get1/2 insertase complex. *B*, *in vitro* reconstitution of the Get3_(D57N)_–Get4/5 complex. Shown are size-exclusion chromatography profiles of Get3_(D57N)_ (*yellow*), Get4/5 (*green*), and the reconstituted Get3_(D57N)_–Get4/5 complex (*purple*). SDS-PAGE analysis of peak fractions confirms the presence of all components in the assembled complex. *C*, cryo-EM density map of the Get3(D57N)–Get4/5 complex, shown in two orthogonal views rotated by 90°. *D*, ribbon diagram of the Get3(D57N)–Get4/5 complex, with Get3, Get4, and Get5 colored as in (*C*). Bound ATP molecules are depicted as space-filling models.

The transfer of a TA from the cytosolic chaperone Sgt2 to the targeting factor Get3 is a key commitment step in the GET pathway, directing the TA to the ER. For Get3 to receive the TA, it must adopt a closed, nucleotide-bound conformation, which forms a hydrophobic chamber capable of accommodating the TA's transmembrane domain. Previous structural studies have shown that Get3 cycles between this closed, nucleotide-bound conformation (with ATP or ADP) and an open, nucleotide-free state (14, 15, 16, 17, 18, 19). In the closed conformation, two Get3 monomers join at the dimer interface to form a hydrophobic TA-binding chamber, lined by residues from both monomers, that accommodates the TA's transmembrane domain (20). According to the current model, the Get4/Get5 complex (hereafter referred to as Get4/5) binds and primes Get3 for TA binding by (1) inhibiting Get3's ATP hydrolysis, thereby locking it in the ATP-bound closed conformation and (2) positioning TA-bound Sgt2 near Get3 to facilitate the TA transfer and prevent loss or aggregation (21). Therefore, Get4/5 is believed to serve the dual role by regulating Get3 conformation and bridging the interaction between Sgt2 and Get3 during this critical TA transfer step.

Despite progress in defining the overall GET pathway, the precise mechanism by which Get4/5 coordinates the protected transfer of TA from Sgt2 to Get3 remains unclear. How Get4/5 inhibits Get3's ATPase activity and positions Sgt2 relative to Get3 to enable protected handoff of the TA without cytosolic exposure not yet fully understood. A crystallographic structure of ATP-bound yeast Get3 in complex with truncated Get4/5 (full-length Get4 and the N-terminal peptide of Get5) revealed that Get4 binds across the Get3 dimer interface near the ATPase domain (22). However, resolution limitations and the use of truncated Get5, lacking its Sgt2-binding domain, left the mechanisms of ATPase inhibition and the spatial positioning of Sgt2 undetermined—both critical for understanding the Get4/5-mediated TA transfer step.

More recently, a high-resolution cryo-electron microscopy (cryo-EM) structure of a metazoan ortholog of Get3 and Get4/5 in complex showed that, in addition to binding across the Get3 dimer interface as previously observed in the yeast Get3-Get4/5 structure, Get4/5 interacts with Get3 to form an apical composite lid over the TA-binding chamber and also engages a region linked to the ATPase domain (23). In the same study, the authors crosslinked Sgt2 to the Get3-Get4/5 complex and observed a low-resolution density near the top of the TA-binding chamber, above the apical lid, which they proposed corresponded to Sgt2 (23). However, the poor local resolution in this region, coupled with the observed density not fully accounting for the size of Sgt2 (likely due to its inherent flexibility), precluded confident model building and left the precise position of Sgt2 uncertain.

While these structural studies have provided valuable mechanistic insights, further investigation is needed to elucidate the molecular basis for Get4/5-mediated inhibition of Get3's ATPase activity and to precisely define the location of the Sgt2-binding domain within the Get3-Get4/5 complex. To address this gap, we determined the high-resolution structure of the intact yeast Get3-Get4/5 complex. Yeast, being the most thoroughly studied for the GET pathway, is the ideal system to explore whether key structural features observed in higher eukaryotes but not yet identified in yeast, such as the composite lid and the region of Get3 adjacent to the ATPase domain that interacts with Get4/5, are conserved.

Here, we report the 3.2 Å cryo-EM structure of the *Saccharomyces cerevisiae* Get3-Get4/5 complex, using full-length Get4/5 containing the Sgt2-binding domain. This structure provides new insights into the interactions between Get4/5 and Get3, while defining the position of the Sgt2-binding domain of Get5 within the complex. Our findings reveal that the yeast Get3-Get4/5 complex shares conserved features such as the composite lid seen in higher eukaryotes, but the Sgt2-binding position appears to differ within the complex, raising new hypotheses regarding the molecular mechanisms governing TA targeting in yeast.

## Results

### Purification and structural determination of the intact yeast Get3-Get4/5 complex

To determine the cryo-EM structure of the intact *S. cerevisiae* Get3-Get4/5 complex, full-length Get3 and Get4/5 proteins were overexpressed and purified separately from *Escherichia coli* before assembly. As previous studies have shown that ATP-bound Get3 exhibits the highest affinity for Get4/5 (22), we utilized the Get3_(D57N)_ variant. This variant carries a point mutation in the catalytic residue D57, which is essential for ATP hydrolysis (24). Mutation of this residue has been previously shown to effectively trap Get3 in its ATP-bound state by allowing ATP binding without subsequent hydrolysis (22).

As reported in previous studies (16, 21, 25), Get3_(D57N)_ was purified in both dimeric and tetrameric forms, whereas Get4/5 was purified as a heterotetramer containing two copies of Get4/5 heterodimer (Fig. S1, *A* and *B*). The Get3_(D57N)_-Get4/5 complex was then formed *in vitro* by mixing equimolar amounts of the purified Get3_(D57N)_ dimer (physiologically relevant oligomeric state) and Get4/5 heterotetramer, followed by further purification of the complex *via* size-exclusion chromatography. The Get3_(D57N)_-Get4/5 complex eluted as a monodisperse peak with a molecular weight of 210 kDa, as measured by SEC-MALS (Size-Exclusion Chromatography coupled with Multi-Angle Light Scattering), consistent with an intact complex consisting of a Get3_(D57N)_ dimer interacting with Get4/5 heterotetramer (Figs. 1*B* and S1*C*). The monodisperse nature of the elution peak and the expected molecular weight confirm that the complex remains intact without any detectable degradation or dissociation of its components, providing a high-quality sample for subsequent cryo-EM analysis.

Following cryo-EM data collection and processing, including multiple rounds of 3D classifications initially without a mask and later with masking, a conformationally homogenous subset of particles was isolated and refined to yield a 3.2 Å reconstruction of the Get3_(D57N)_-Get4/5 complex (Figs. 1, *C* and *D*, S1, S2 & Table S1). While the overall complex was well-resolved, with clear density for most amino acid side chains, certain regions exhibited lower local resolution. For example, the composite lid formed by Get3 and Get4 showed lower local resolution due to flexibility (Fig. S3*D*), but these were effectively modeled using structures predicted by ColabFold (26). Notably, the Sgt2-binding domain of Get5, initially unresolved from the high-resolution map, was recovered through 3D classification (Fig. S2) and modeled using a predicted domain structure (Fig. 5, *A* and *B*), as described later. In contrast, despite the presence of intact proteins as confirmed by SDS-PAGE analysis (Fig. 1*B*), the C-terminal region of Get5 containing the dimerization domain (residues 153–212) remained invisible, reflecting its dynamic nature. Our final model of the Get3_(D57N)_-Get4/5 complex encompasses nearly the full sequence of Get3_(D57N)_ and Get4, and Get4-and Sgt2-binding domains of Get5 (Figs. 1*D* and 5, *A* and *B*), representing the most complete structure of the yeast Get3-Get4/5 complex to date.

### Novel interaction interfaces in the yeast Get3-Get4/5 complex

**Figure 2:**
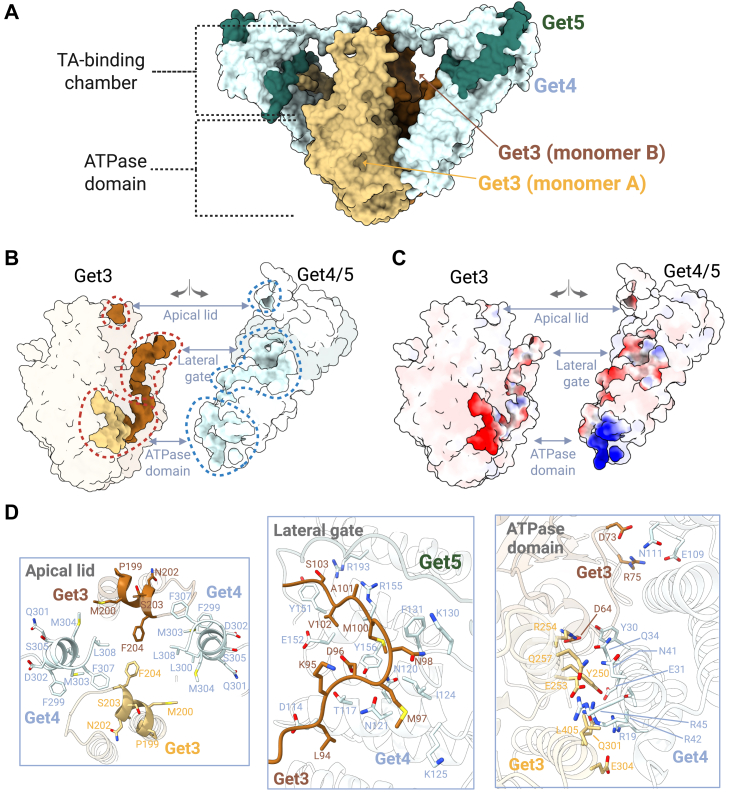
**Interaction interface between Get3 and Get4/5.***A*, domain organization within the Get3_(D57N)_–Get4/5 complex. The ATPase-binding domain is formed entirely by Get3, while the TA-binding chamber in formed by Get3 and Get4/5. *B*, three distinct interfaces mediate the interaction between Get3 and Get4/5. Contact regions between the proteins are highlighted in *solid* (*non-faded*) colors, using the same color scheme as in (*A*). *C*, electrostatic surface representation of the contact interfaces between Get3 and Get4/5 within the Get3_(D57N)_–Get4/5 complex. *D*, close-up of the three interaction interfaces between Get3 and Get4/5.

**Figure 3:**
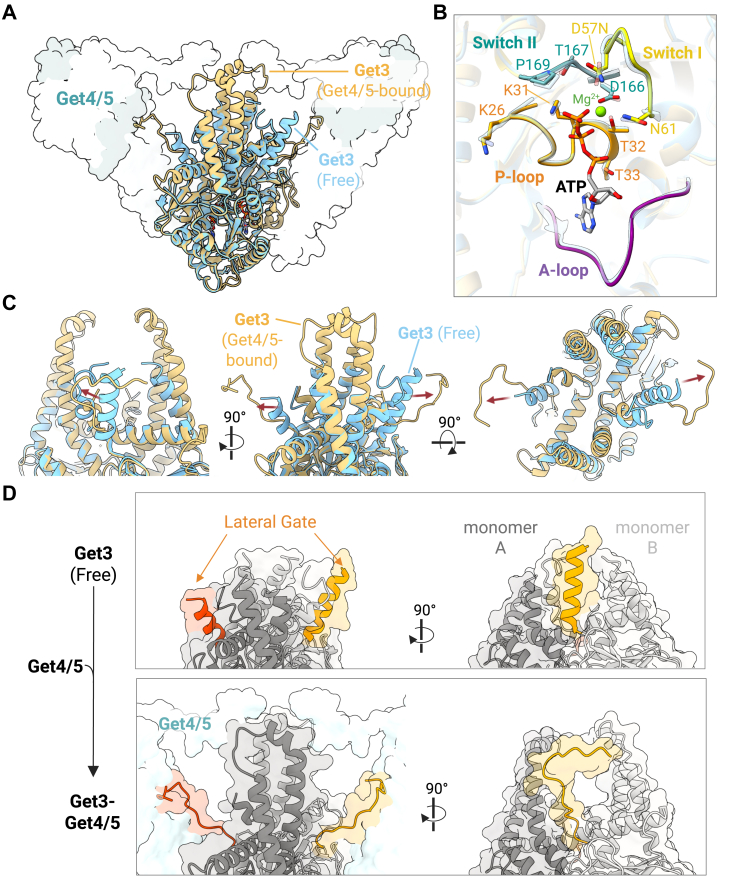
**Structural remodeling of Get3's TA-binding chamber upon Get4/5 binding.***A*, overlay of Get3 from the Get3_(D57N)_–Get4/5 complex (*yellow*) with Get3 alone (*blue*; PDB 2WOJ). Get4/5 is shown as a transparent surface. *B*, comparison of the catalytic site architecture in the ATPase domain of Get3 from the Get3_(D57N)_–Get4/5 complex (*yellow*) and from Get3 alone (*blue*). Key motifs within the ATPase domain are highlighted in *solid colors*: switch I (*yellow*), switch II (*cyan*), P-loop (*orange*), and A-loop (*purple*). *C*, overlay of the TA-binding chamber of Get3 from the Get3(D57N)–Get4/5 complex (*yellow*) and Get3 alone (*blue*), highlighting a helix-to-coil transition in the lateral wall upon Get4/5 binding. *D*, lateral opening of the TA-binding chamber upon Get4/5 binding. In the absence of Get4/5 (*upper panel*), helices forming the lateral wall of the substrate-binding chamber (*red* and *yellow*) remain folded and closely packed. Upon Get4/5 binding (*lower panel*), these helices partially unfold and shift outward, resulting in a more exposed and accessible lateral gate of the TA-binding chamber.

Our cryo-EM structure of the yeast Get3_(D57N)_-Get4/5 complex recapitulates the previously established overall architecture while revealing interaction interfaces that were unresolved in earlier studies. The complex adopts a two-fold symmetric arrangement, with a central Get3_(D57N)_ dimer in an ATP-bound closed conformation (Figs. 1*D* and 3*B* & S3*E*), flanked by two opposing Get4/5 heterodimer arms extending outward (Fig. 1, *C* and *D*). Each Get3_(D57N)_ monomer engages a Get4/5 heterodimer through three distinct interaction surfaces, mirrored symmetrically on the opposite side of the dimer, resulting in six contact points across the assembly (Fig. 2). These interfaces are distributed across the surfaces of Get3's ATPase domain and TA-binding chamber, including one at the ATPase domain and two at the TA-binding chamber—the latter representing interaction sites not previously resolved in yeast Get3-Get4/5 structures. The corresponding interaction sites on Get4/5 span along its extended structure (Fig. 2, *B*–*D*).

The first interface, involving the ATPase domain of Get3 and the N-terminal region of Get4, is consistent with prior structural studies of the yeast complex (22) and features a combination of hydrophobic and electrostatic interactions (Figs. 2, *B*–*D* & S7). In this arrangement, Get4 from one Get4/5 arm interacts with both Get3 monomers near their ATPase domains, burying approximately 886 Å^2^ of surface area.

The second and third interfaces are newly resolved in our structure and involve regions of the TA-binding chamber that were previously unresolved in yeast Get3-Get4/5 structures. The second interface involves a lateral region of Get3's TA-binding chamber we term the “lateral gate,” composed of hydrophobic and charged amino acids (residues 94–104) in Get3 (Fig. 2, *B*–*D*). This site is particularly notable as the only region in the complex where Get3, Get4, and Get5 directly contact one another. Here, Get3 simultaneously engages both Get4 and Get5, burying roughly 721 Å^2^ of surface area (Fig. 2*D*).

The third interface is created by a conserved amphipathic α-helix (helix 8; residues 198–204) positioned in the apical surface, or “lid”, of Get3's TA-binding chamber (Fig. 1*D*). This helix interacts with the amphipathic C-terminal α-helix of Get4 (residues 299–308), which extends from the distal tip of the Get4/5 arm toward the lid region of Get3. Together, these helices form a composite lid over the TA-binding chamber, with their hydrophobic faces oriented toward the chamber interior. This interaction buries approximately 100 Å^2^ of surface area (Fig. 2*D*).

While the interaction between Get3's ATPase domain and Get4 has been previously described (22), the two TA chamber-associated interfaces (*i.e.*, the lateral gate and the composite lid) were not resolved in earlier *S. cerevisiae* Get3-Get4/5 complex structures, likely due to conformational flexibility in these regions (Fig. S7). Similar features, however, have been observed in the metazoan complex formed by Get3 and Get4/5 orthologs (Fig. S7) (23), suggesting a conserved structural features that had not been captured in yeast until now.

### Get4/5 binding drives structural changes in Get3's TA-binding chamber without altering the ATPase domain conformation

Earlier studies proposed that Get4/5 primes Get3 by inhibiting its ATPase activity, stabilizing it in an ATP-bound closed conformation necessary for TA capture (21). Consistent with this model, our Get3_(D57N)_-Get4/5 structure shows Get3 in the expected closed, ATP-bound conformation. However, comparison with prior Get3 structures lacking Get4/5 (*e.g.*, PDB: 2WOJ) shows that the ATPase domain itself remains largely unchanged upon Get4/5 association (Fig. 3, *A* and *B*). This suggests that the previously reported decrease in Get3's ATP hydrolysis rate upon Get4/5 interaction does not result from altering the catalytic site, but rather through other mechanisms, likely by stabilizing the closed conformation in which the nucleotides are buried (Fig. 1*D* and S3*E*) and less able to readily dissociate.

Key catalytic and nucleotide-coordinating elements in the ATPase domain, including P-loop residues K26, T32, and T33, as well as the catalytic residue D57 (mutated to N in this study), adopt conformations compatible with ATP binding and hydrolysis, indistinguishable from those observed in Get3 alone (Fig. 3, *A* and *B*). Root mean square deviation (RMSD) analysis further confirms this structural preservation, with minimal deviation across the ATPase domain backbone (RMSD = 0.5 Å).

In contrast, notable structural rearrangements occur within Get3's TA-binding chamber upon Get4/5 engagement (Fig. 3, *C* and *D*). Specifically, the chamber adopts a more open conformation, with its lateral wall becoming fully exposed—a marked change from the more enclosed (but not fully sealed) state observed in Get3 structures lacking Get4/5. This opening is driven by a helix-to-coil transition in residues 94 to 104 of each Get3 monomer. In the absence of Get4/5, these residues form an α-helix, contributing to the lateral walls of the chamber. Upon Get4/5 binding, they lose their helical structures, unfold, and reposition outward to engage with Get4/5, creating a novel interface (Figs. 2*D* and 3, *C* and *D*). This structural rearrangement increases the solvent-accessible surface area of the chamber (from 1394 Å^2^ to 1625 Å^2^), effectively opening the lateral gate, possibly for increased substrate access.

The apical lid of the TA-binding chamber also undergoes structural changes upon Get4/5 binding. In the absence of Get4/5, this region of Get3, formed by helix 8, is typically flexible and often unresolved. Upon Get4/5 binding, however, it becomes partially ordered through its interaction with the C-terminal helix of Get4, together forming the composite lid described earlier (Fig. 2*D*). Despite this interaction, it seems that the lid region remains relatively flexible, as indicated by lower local resolution in this area (Figs. 1, *C* and *D* and S3*D*).

In summary, these findings highlight that while Get4/5 binding does not reconfigure the catalytic core and overall structure of Get3's ATPase domain, it induces significant remodeling within the TA-binding chamber. Specifically, the lateral walls open through a helix-to-coil transition stabilized by interaction with Get4/5, while the chamber's lid also engages with Get4/5, becoming more ordered yet retaining flexibility.

### Intrinsic structural flexibility of Get3's TA-binding chamber

Building on the observed flexibility of the lateral gate and apical lid of Get3 in our cryo-EM structure, we sought to gain insight into whether Get3's TA-binding chamber possesses intrinsic flexibility that facilitates the structural rearrangements induced by Get4/5 binding, as observed in our cryo-EM structure.

**Figure 4:**
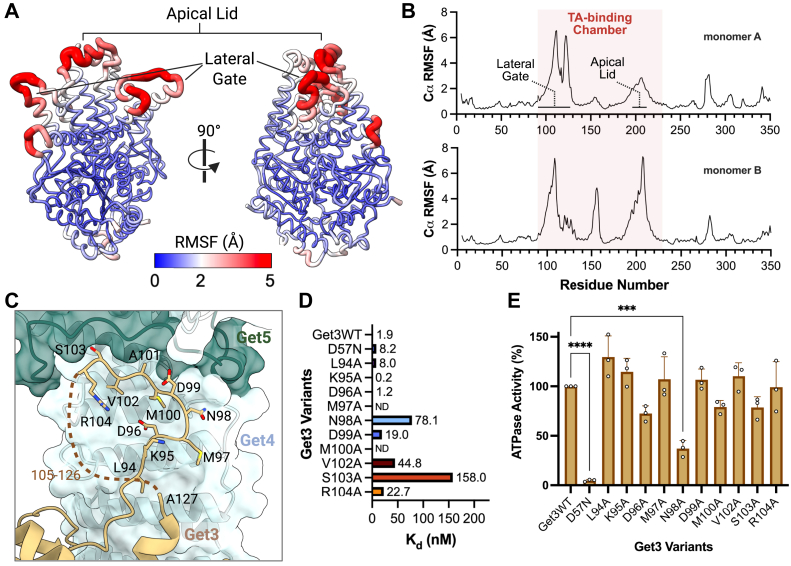
**Structural dynamics and functional analysis of lateral gate residues.***A*, root meansquare fluctuation (RMSF) analysis of Get3, shown as worm representations colored by RMSF values from molecular dynamics simulations (*blue* = low, *red* = high flexibility). The apical lid and lateral gate regions display the highest flexibility. *B*, RMSF profiles for each Get3 monomer, highlighting the TA-binding chamber (*shaded red*), with peaks corresponding to the lateral gate and apical lid regions. *C*, detailed view of Get3's lateral gate residues (L94–R104) at the interface with Get4 and Get5. Individual residues shown were systematically mutated to alanine to assess their functional importance in Get4/5 binding and ATPase activity (data shown in *panelsD* and *E*). *D*, binding affinities (K_d_) of Get3 variants for Get4/5, measured by microscale thermophoresis (MST). ND indicates that the binding affinity could not be determined. *E*, relative ATPase activity of Get3 variants, reported as a percentage of wild-type (WT) activity. The D57N variant, used as a negative control, shows minimal activity, while other mutations at the lateral gate variably affect ATPase function. Statistical significance was assessed using one-way ANOVA followed by Dunnett's *post hoc* test. ∗∗∗∗*p* < 0.0001; ∗∗∗*p* < 0.001. Each data point represents a technical replicate (n = 3 assay replicates per variant).

To assess flexibility in the absence of Get4/5, we performed all-atom molecular dynamics (MD) simulations of Get3 in its closed, ATP-bound conformation. These simulations revealed that while the ATPase domain remains highly stable, the TA-binding chamber exhibits significant flexibility. The lateral gate and helix 8, which forms the chamber's apical lid, showed the highest atomic fluctuations (Fig. 4, *A* and *B*), suggesting that these regions of the TA-binding chamber are inherently flexible and likely prone to undergoing conformational changes.

We then examined the Get3_(D57N)_-Get4/5 cryo-EM map to gain insight into the structural dynamics after Get4/5 binding. Visual inspection of the map, combined with a local resolution heatmap, revealed that the ATPase domain of Get3, along with Get4 and the segment of Get5 interacting directly with Get4, were highly ordered, with resolution better than 3 Å (Fig. S3*D*). The lateral gate residues, which undergo the helix-to-coil transition, were also well ordered (3.5 Å or better) (Fig. S3*D*), indicating this region becomes more ordered upon Get4/5 binding, despite losing its secondary structure. In contrast, the apical lid, while more ordered compared to free Get3 structures, remained more flexible than the lateral gate, as reflected by lower local resolution (3.5 Å or worse) in this region (Fig. S3*D*). Thus, the lateral gate seems to undergo a shift from a flexible α-helix to a more ordered coil state upon engagement with Get4/5, whereas the apical lid retains some flexibility, although more ordered.

**Figure 5:**
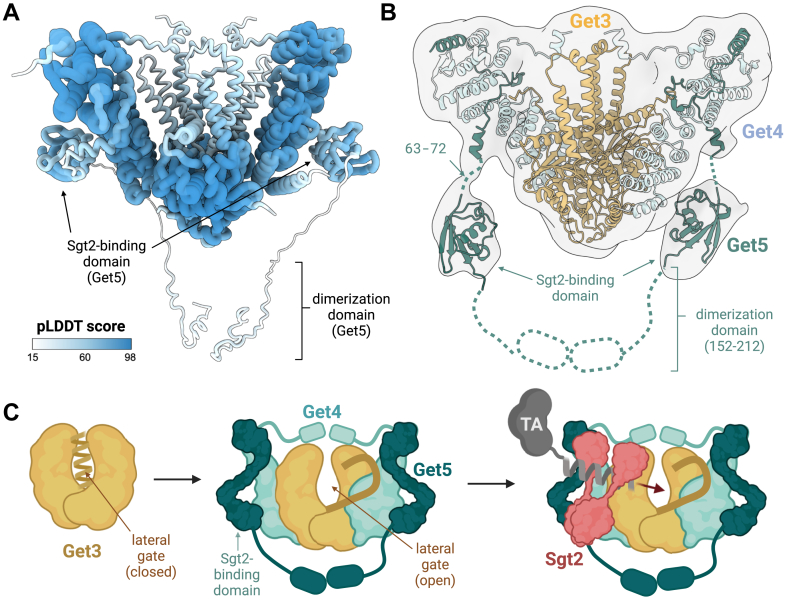
**Mapping the Sgt2-binding domain within the Get3_(D57N)_-Get4/5 complex.***A*, ColabFold prediction of the Get3-Get4/5 complex, colored by pLDDT values and displayed as a coil representation. Thicker regions indicate higher prediction confidence (higher pLDDT values). *B*, fitting of the Sgt2-binding domain of Get5 into newly identified density in the low-resolution cryo-EM map obtained after 3D classification. *C*, proposed model for TA substrate transfer from Sgt2 to Get3. Before Get4/5 binding, the lateral gate region of Get3 adopts a helical conformation. Upon Get4/5 binding, this region undergoes a helix-to-coil transition, resulting in opening of the lateral gate and increased accessibility of the TA-binding chamber. The proximity of the Sgt2-binding domain of Get5 to the opened lateral gate enables recruitment of the Sgt2-TA complex and facilitates lateral transfer of the TA substrate to Get3.

To further explore the flexibility of the Get3-Get4/5 complex, we used ColabFold (26) to generate multiple predicted structural models and compared the alignment of various regions across these predictions. The rationale was that stable, well-defined regions would display high structural similarity between models, while more flexible regions would show greater variability. Consistent with our cryo-EM and MD findings, the ATPase domain of Get3 exhibited high structural concordance across ColabFold models, with pairwise RMSD values ranging from 0.5 to 1.9 Å and mean pLDDT scores (a per-residue confidence metric) between 84 and 88 (Figs. 5*A* & S6). In contrast, the TA-binding chamber of Get3 and the C-terminal helix of Get4 interacting with it exhibited substantial conformational variability, with RMSD values ranging from 1.1 to 3.3 Å and lower mean pLDDT scores between 48 and 62 (Figs. 5*A* & S6). It should be noted that none of the models correctly predicted the unfolding of the lateral gate in the Get4/5-bound form, which may explain the observed high variability in the lateral gate residues. Nevertheless, the overall trend of a stable ATPase domain and a more flexible TA-binding chamber aligns with our cryo-EM and MD results.

Together, these computational and structural analyses suggest that Get3's TA-binding chamber is intrinsically dynamic, with its lateral gate particularly predisposed to structural reconfiguration upon Get4/5 binding.

### Structural and functional roles of Get3's lateral gate residues

To investigate the structural and functional significance of the region that undergoes helix-to-coil transition upon Get4/5 binding, we generated a series of Get3 variants targeting residues within this region (residues 94–104), which we refer to as the lateral gate (Fig. 4*C*). Single-site alanine substitutions were introduced at each position within this segment. All variants expressed and purified similarly to wild-type Get3, with comparable yields, purity, and monodisperse behavior by size-exclusion chromatography, indicating that these mutations did not disrupt overall folding (Fig. S4).

Since the lateral gate residues directly interacts with Get4/5 in our cryo-EM structure and Get3's interaction with Get4/5 is essential for TA transfer from Sgt2 (20), we examined how these mutations affected Get3's affinity for Get4/5. Using microscale thermophoresis (MST) (27), we measured the binding affinities of wild-type Get3, the ATPase-deficient D57N variant, and the lateral gate mutants for Get4/5(Fig. 4*D*). Wild-type Get3 and the D57N variant bound Get4/5 with dissociation constants (K_d_) of 1.9 nM and 8.2 nM, respectively. Several mutations within the lateral gate region significantly weakened this interaction, reducing binding affinity by 10-fold or more. These included D99A (K_d_ = 19.0 nM), N98A (K_d_ = 78.1 nM), V102A (K_d_ = 44.8 nM), S103A (K_d_ = 158 nM), and R104A (K_d_ = 22.7 nM). Notably, these residues cluster near the interface where Get3, Get4, and Get5 converge, suggesting a key role for this region in complex formation or stabilization (Fig. 4*C*). Among these, the N98A and S103A exhibited the most pronounced effects, with S103A reducing affinity by over 80-fold relative to wild-type (Fig. 4*D*).

To determine whether these mutations also impacted Get3's enzymatic function, we measured the ATPase activity of each variant (Fig. 4*E*). Most substitutions had little to no effect on the ATPase activity, indicating that these mutations primarily influence Get3-Get4/5 binding rather than ATP turnover. However, the N98A mutation resulted in a marked reduction in ATPase activity, retaining less than half the activity of wild-type Get3 (Fig. 4*E*). Although N98 is spatially distant from the ATP-binding site, its position within the lateral gate, a region structurally connected to the ATPase domain, raises the possibility of an allosteric role, though the underlying mechanism remains unclear. Interestingly, as mentioned above, N98A also caused one of the largest decreases in Get4/5 binding affinity (∼40-fold), underscoring this residue as a functionally important site that couples Get3's ATPase activity with its interaction with Get4/5.

### Mapping the Sgt2 recruitment site within the Get3_(D57N)_-Get4/5 complex

A critical step toward understanding how Get4/5 facilitates TA transfer from Sgt2 to Get3, while shielding the TA from the cytosol, is identifying the location of Get5's Ubl (ubiquitin-like) domain (residues 73–149). This domain, known to serve as the recruitment site for Sgt2 (28), is expected to be positioned near Get3's TA-binding chamber to minimize loss and aggregation, although the precise route for TA handoff remains unresolved.

In our initial high-resolution cryo-EM map, we successfully modeled density corresponding to Get5 residues 7 to 53. However, the remainder of Get5's C-terminal portion (residues 54–212), containing both the Ubl and dimerization domains, was unresolved (Fig. 1, *C* and *D*). To investigate whether structural heterogeneity might account for this missing region, we performed 3D classification analysis on our cryo-EM data to identify conformations containing additional ordered features. This analysis revealed a new and consistent density feature present across multiple classes (Fig. S2), extending from Get5 residue 53 (the most C-terminal residue we could initially model) and is positioned adjacent to Get3's TA-binding chamber, near the lateral gate opening (Fig. 5, *A* and *B*).

Although the resolution of this region was insufficient for confident atomic modeling, the shape and volume of the newly observed density (4147 A^3^) closely matched the estimated size and shape of Get5's Ubl domain (4122 A^3^) predicted by our ColabFold model (Figs. 5, *A* and *B* & S6*A*). Based on this correlation and the consistent spatial location of the feature, we propose that this density likely corresponds to the Get5's Ubl domain.

To the best of our knowledge, this represents the first structural insight into the location of the Get5's Ubl domain within the Get3-Get4/5 complex. While further structural and biochemical validation will be required, our model provides important spatial context for understanding how Sgt2 is recruited to the complex and how its TA cargo might be handed off to Get3.

## Discussion

In this study, we determined the cryo-EM structure of the *S. cerevisiae* Get3-Get4/5 complex, providing new insights into how Get4/5 primes Get3 to receive a TA substrate from Sgt2 during the early steps of the GET pathway. Our structure shows that Get4/5 binding induces substantial remodeling within Get3's TA-binding chamber, resulting in lateral openings that could enhance substrate accessibility. Additionally, we mapped the Sgt2-binding site within the complex, offering clues about how Get4/5 might spatially organize Sgt2 and Get3 to coordinate direct TA handoff.

### Get4/5's role in priming Get3 for TA binding

Get4/5 is proposed to facilitate TA transfer from Sgt2 to Get3 by stabilizing Get3 in an ATP-bound, closed conformation that is optimal for TA binding, by inhibiting ATP hydrolysis. The structural basis for this inhibition, however, has been unclear.

In our cryo-EM structure of the Get3_(D57N)_–Get4/5 complex, Get3 adopts an ATP-bound, closed conformation. Interestingly, the catalytic residues in Get3's ATPase domain are unchanged compared to previously solved ATP-bound structures lacking Get4/5 (Fig.3, *A* and *B*) (14, 15, 17, 18, 20). Therefore, the previously reported inhibition of Get3's ATP hydrolysis by Get4/5 (21) does not seem to result from direct changes to the catalytic site, but may instead occur through indirect effects on the dimer interface, where Get4/5 binding could impede nucleotide exchange.

Unlike the ATPase domain that remains unchanged, Get4/5 binding induces structural rearrangements in the TA-binding chamber of Get3. Most notably, a region we designate as the lateral gate, which forms part of the wall of the TA-binding chamber and adopts an α-helical conformation in free Get3, undergoes a helix-to-coil transition upon Get4/5 binding (Fig. 3, *C* and *D*). In our structure, this region is unstructured and engaged by Get4/5, creating two lateral openings on opposite sides of the chamber that increase solvent and presumably substrate accessibility. At the same time, the apical surface of the TA-binding chamber, which is typically disordered and unresolved in structures lacking Get4/5, becomes more stable and interacts with a C-terminal α-helix from Get4 to form a composite lid over the chamber. However, this region still seems to retain conformational flexibility, as indicated by its low local resolution in the cryo-EM map (Fig. S3*D*).

These structural changes in the TA-binding chamber were not visible in previously published yeast Get3-Get4/5 crystal structures (22, 29). However, they closely resemble those observed in the metazoan Get3-Get4/5 complex, where Get3 undergoes a similar helix-to-coil transition upon Get4 binding (Fig. S7). Interestingly, the residues in the lateral gate that undergo conformational changes are divergent in sequence between yeast and higher eukaryotes (23). However, the similar structural behavior in yeast, despite the poor sequence similarity, suggests that this is a conserved mechanism across species, with both yeast and higher eukaryotes using a common strategy to modulate TA-chamber accessibility.

The precise functional role of the lateral gate remains to be fully defined, but we hypothesize that its effect of opening the TA-binding chamber likely plays a key role in increasing TA-substrate accessibility. In free Get3, the lateral gate predominantly exists as a flexible α-helix, which, while not fully occluding the TA-binding chamber, likely restricts lateral access and limits the chamber's openness. Upon Get4/5 binding, Get4/5 unfolds and sequesters the lateral gate away from the chamber opening, effectively holding the gate open. This increases solvent exposure and accessibility of the hydrophobic cleft for incoming TA substrates, a remodeling mechanism likely critical for efficient substrate capture during the handoff from Sgt2.

Additionally, our mutagenesis data suggests that the lateral gate may play a dual role, influencing both substrate accessibility and ATPase regulation. A mutation at N98, within the lateral gate, significantly reduced ATPase activity (Fig. 4*E*), hinting at possible allosteric communication between the chamber region and the ATPase site. A similar hypothesis was suggested in the metazoan study (23), where the lateral gate was proposed to coordinate both substrate binding and ATP hydrolysis. These findings raise the intriguing possibility that the lateral gate coordinates the regulation of both substrate binding and ATP hydrolysis, although further studies are needed to fully explore this hypothesis.

### Get4/5's role in Sgt2 recruitment and TA handoff

To prevent the loss and aggregation of TAs during transfer, a precisely coordinated direct handoff is likely required, with Get4/5 bridging the interaction between Sgt2 and Get3. However, the exact mechanisms by which Get4/5 facilitates this interaction remain unclear, partly due to limited structural information on the Sgt2 binding domain of Get5, which was either truncated or unresolved in previous structural studies of the yeast Get3-Get4/5 complex.

A recent cryo-EM study of zebrafish Get3 in complex with human Get4/5 and SGTA (the mammalian homolog of Sgt2), stabilized by crosslinking, revealed additional density near the apical lid of Get3's TA-binding chamber (23). This density was attributed to SGTA and formed the basis for a model in which SGTA delivers the TA to the apical surface of Get3, promoting apical lid opening and allowing the substrate to slide downward toward the hydrophobic cleft at the bottom of the chamber. While these findings offer valuable mechanistic insights, the low resolution of the observed density prevented definitive assignment to a specific SGTA domain, leaving the precise route and orientation of TA handoff open for further investigation.

In our cryo-EM structure of the yeast Get3-Get4/5 complex, we identified a consistent density feature adjacent to the lateral gate of Get3, extending from the last modeled C-terminal residue of Get5. Although the resolution of this feature precludes atomic modeling, its size, shape, and location are consistent with the predicted dimensions of the Get5 Ubl domain based on ColabFold models (Figs. 5, *A* and *B* & S6*A*). Based on this positioning of the Ubl domain, we propose an alternative model in which TA delivery occurs through a lateral entry pathway rather than through the apical lid (Fig. 5*C*). In this model, Get4/5 promotes the opening of the lateral gate in Get3 through a helix-to-coil transition, while positioning Sgt2 nearby *via* the Ubl domain. This spatial arrangement could allow the TA to be handed off directly into the hydrophobic cleft of Get3 through the lateral gate, minimizing cytosolic exposure during the transfer.

This proposed lateral handoff route differs from the apical model proposed by the previous study of the metazoan Get3-Get4/5 complex (23). While the discrepancy may reflect species-specific differences or the influence of crosslinking in the metazoan study, we acknowledge that the position of the Ubl domain may shift upon Sgt2 binding, and that its proximity to the lateral gate in our structure does not exclude the possibility of Sgt2 engaging the apical lid. Additionally, the lateral opening observed in our structure could serve a different function entirely, such as providing space to accommodate the incoming TA substrate after initial docking through the apical surface. Thus, while our model offers a plausible mechanistic framework, it remains speculative. Further structural and biochemical work will be essential to test both lateral and apical handoff models and determine whether they represent distinct functional states, coexisting pathways, or species-specific adaptations.

In addition to spatial organization, the thermodynamics of substrate binding likely also contribute to the directionality and efficiency of TA handoff. Specifically, a hydrophobicity gradient between the binding sites of Sgt2 and Get3 may help drive the transfer once lateral access is available. Sgt2's substrate-binding domain is predicted to be less hydrophobic and binds TA with lower affinity than the deeply hydrophobic cleft of Get3's TA-binding chamber (20, 30, 31). This could serve as a thermodynamic driver for the unidirectional transfer of TA from Sgt2 to Get3.

### Future directions

Several important questions remain regarding the early steps of the GET pathway. On the structural front, capturing the interaction between Get3-Get4/5 and Sgt2, either in isolation or in complex with TA substrates, would provide a critical next step toward assessing the mechanism of TA handoff. High-resolution structures of these complexes could reveal how Sgt2 engages with the Get4/5 complex, how the TA is positioned relative to Get3's lateral gate or apical lid, and whether specific conformational changes in the machinery accompany substrate transfer.

Functionally, the role of the dynamic lateral gate in Get3 merits deeper investigation. While our findings suggest that lateral gate opening could plausibly provide an entry route for TA substrates, its specific role in TA handoff remains untested. Additional biochemical and structural analyses using lateral gate mutants in combination with TA transfer and membrane insertion assays will be important to define whether and how this feature contributes to substrate handoff and downstream targeting steps.

Additionally, testing the hydrophobicity gradient hypothesis—through *in vitro* transfer assays with mutant chaperone variants of altered hydrophobic character, or through molecular dynamics simulations modeling substrate transfer —may offer valuable insights into the thermodynamic principles that drive TA handoff, irrespective of whether lateral or apical entry is used.

We hope this work helps generate new hypotheses and lays a foundation for future studies exploring the intricacies of TA protein targeting and the broader principles of chaperone-mediated membrane protein biogenesis.

## Experimental procedures

### Constructs

To generate the constructs for expressing either Get3 or Get3_(D57N)_, the corresponding *S. cerevisiae* gene was cloned into the pET28a vector with an N-terminal His_6_ tag. For expressing the Get4/5 complex, the genes encoding Get4 (with an N-terminal His_6_ tag) and Get5 (untagged) were cloned into the pRSF-Duet vector (Novagen) for co-expression. These constructs also served as templates for introducing single-point mutations in Get3 and Get4 using the QuickChange II site-directed mutagenesis kit (Agilent).

### Expression and purification of Get3, Get3_(D57N)_, and Get4/5

Get3, Get3_(D57N)_, and Get4/5 were expressed and purified using the same procedure. Proteins were overexpressed in Rosetta (DE3) (Millipore Sigma) cells grown in LB medium at 37 °C until reaching an optical density value of 0.6 at 600 nm. Protein expression was induced with 1 mM isopropyl β-D-1-thiogalactopyranoside (IPTG) for 3 h. Following induction, cells were harvested by centrifugation at 4,000*g* for 20 min. The resulting bacterial cell pellets were lysed using sonication, and the lysates were ultracentrifuged at 30,000*g* for 30 min. The clarified lysates were loaded onto a Ni-NTA column, and the resin was washed with a buffer containing 50 mM HEPES (pH 7.5), 500 mM NaCl, 1 mM DTT, and 50 mM imidazole to remove nonspecifically bound proteins. The target proteins were then eluted using a buffer of the same composition but with 200 mM imidazole. The elution fractions from the Ni-NTA purification were further purified by size-exclusion chromatography on a Superdex 200 Increase 10/300 Gl column equilibrated with a buffer consisting of 20 mM HEPES (pH 7.5), 150 mM NaCl, and 1 mM DTT. Protein-containing fractions were collected, aliquoted, and stored at −80 °C for later use.

### Assembly and purification of the Get3-Get4/5 complex

To assemble the Get3_(D57N)_-Get4/5 complex *in vitro,* purified Get3_(D57N)_ and Get4/5 were mixed in an equimolar ratio to a final concentration of 4 μM. ATP and MgCl_2_ were added to the mixture to a final concentration of 2 mM. The reaction was incubated overnight (∼12 h), after which the mixture was loaded onto a Superdex 200 Increase 10/300 Gl column. Peak fractions corresponding to the Get3_(D57N)_-Get4/5 complex were collected, aliquoted, and stored at −80 °C for future use.

### Cryo-EM sample preparation and data collection

Purified Get3_(D57N)_-Get4/5 complex samples were concentrated to a final concentration of 6.0 mg/ml. Immediately before grid preparation, ATP and MgCl_2_ were replenished to 2 mM. Initial cryo-EM trial revealed preferred orientation of the proteins on cryo-EM grids; therefore, n-Dodecyl-β-Maltoside (DDM) was added to a final concentration of 0.05% (v/v) to mitigate this issue. A 4 μl aliquot of the sample was applied to Quantifoil R1.2/1.3400-mesh Cu holey carbon grids. The grids were plunge-frozen using a Vitrobot Mark IV (blot time: 3.5 s, wait time: 10 s, blot force: −2). Data collection was performed on a Titan Krios microscope operating at 300 kV at the Pacific Northwest Cryo-EM Center (PNCC). A total of 5571 movies, each comprising 40 frames, were collected using a Gatan K3 direct electron detector. Images were acquired with a pixel size of 1.0125 Å, a total dose of ∼40 e/Å^2^, and a defocus range of −0.8 μm to −2.8 μm.

### Cryo-EM data processing and model building

The initial 5571 movie frames were aligned and corrected for beam-induced motion using patch motion correction in CryoSPARC (32). The contrast transfer function (CTF) parameters were determined using patch CTF estimation in CryoSPARC, and micrographs with CTF fit parameters worse than 11.562 Å or those showing ice contamination were discarded. A total of 4986 micrographs remained for downstream processing.

Following particle picking and two rounds of 2D classification in CryoSPARC to remove poor-quality particles, 392,467 particles were selected for the multiclass *ab initio* model reconstruction algorithm in CryoSPARC. The best class, containing 267,815 particles, was refined using non-uniform refinement in cryoSPARC (33) before importing the particles into Relion 4.0 (34) for two rounds of 3D-classification. The best 3D class, consisting of 149,868 particles, was reimported to cryoSPARC for a final found of non-uniform refinement, yielding a 3.3 Å map based on the gold-standard FSC.

For atomic model building, the ColabFold-predicted Get3-Get4/5 structure was rigid-body docked into the cryo-EM map using UCSF ChimeraX (35). The model was refined using the real-space refinement algorithm in PHENIX and further adjusted manually in COOT (36). To optimize the overall model geometry and fit to the experimental cryo-EM map, iterative cycles of real-space refinement in PHENIX and manual adjustments in COOT were performed. The stereochemistry and geometry of the structure were assessed using MolProbity (37), and the figures were created using UCSF ChimeraX.

### Structure prediction

The structure of the Get3-Get4/5 complex was predicted using a local implementation of ColabFold 1.3 to serve as an initial model for model refinement, as well as to visualize missing domains not resolved in the cryo-EM map. Sequences for *S. cerevisiae* Get3, Get4, and Get5 were obtained from Uniprot (accesion codes: Q12154, Q12125 and Q12285). An input file specifying a stoichiometry of two copies for each protein in the complex was generated, and the AlphaFold multimer model was used with three recycles and templates to predict five atomic models. Each model was subsequently relaxed using the GPU implementation of Amber. Per-residue pLDDT values were extracted using ChimeraX, and the data were processed and visualized in R.

### MD simulation

To perform molecular dynamics (MD) simulations of Get3 in a closed ATP-bound state, we used the *S. cerevisiae* Get3 structure bound to ADP-AIF_4_ (PDB ID: 2WOJ) as the starting model. Missing residues were grafted from an open-state Get3 structure (PDB ID: 3H84) using convpdb.pl from the MMTSB toolset, and ADP-AIF_4_ was replaced with ATP. To ensure proper zinc coordination, cysteine residues at the dimer interface (C285 and C288 in each monomer) were deprotonated.

A protein structure file (PSF) was generated using genPSF.pl from the MMTSB toolset (38) and used as input to construct a simulation box with CHARMM-GUI v3.7 (39). The system was solvated in a 110 Å cubic box containing explicit TIP3P water and Na^+^/Cl^-^ counterions at 0.15 M. Simulations were performed in OpenMM (40) using the CHARMM36m (41) force field at 303.5 K. After energy minimization (5000 steps), the system was equilibrated in the NVT ensemble for 125 ps before transitioning to the NPT ensemble for a 200 ns production run. Temperature was controlled *via* Langevin dynamics, while pressure was maintained using a Monte Carlo barostat. A soft cut-off of 1.0 nm and a hard cut-off of 1.2 nm were applied for non-bonded interactions, and long-range electrostatics were computed using the particle mesh Ewald (PME) method in OpenMM. Bond lengths involving hydrogen atoms were constrained, and a 2 fs integration time step was used for both equilibration and production.

Root mean square fluctuation (RMSF) plots from the MD trajectory were generated using MDAnalysis 2.8.0 (42, 43) and visualized in UCSF ChimeraX.

### Protein–protein interaction analysis using MST

To determine the equilibrium dissociation constant, K_d_, between Get4/5 and the Get3 variants, we performed microscale thermophoresis (MST) using the Monolith device (NanoTemper Technologies). His-tagged Get4/5 was labeled with 50 nM RED-tris-NTA and maintained at a fixed concentration of 50 nM. Each Get3 variant was subjected to a 12-step, two-fold serial dilution, spanning concentrations from 1000 nM to 0.24 nM. Dilutions were prepared in a buffer containing 20 mM HEPES (pH 7.5), 150 mM NaCl, 1 mM DTT, 2 mM ATP, and 2 mM MgCl_2_. The labeled Get4/5 was incubated with each Get3 concentration, and normalized fluorescence changes were recorded over a 2.5 s interval at 100% laser intensity. Raw MST traces were analyzed using MO Analysis software to determine the Kd. Data for each variant were normalized using a fraction-bound scheme, and affinity curves were compared using MO Analysis.

### NADH-coupled ATPase assay

ATP hydrolysis by different Get3 variants were measured by an enzyme-coupled assay. The reaction mixture contained 20 mM HEPES (pH 7.5), 150 mM NaCl, 1 mM DTT, 5 mM MgCl_2_, 5% Glycerol, 0.3 mM NADH, 10 mM phosphoenolpyruvic acid, 8 units/ml of lactate dehydrogenase, and 6.3 units/ml of pyruvate kinase. ATP hydrolysis coupled to the oxidation of NADH was monitored in real-time for 1 h by measuring absorbance at 340 nm at 30 ˚C using a microplate reader (SpectraMax iD5; Molecular Devices). The data collected were used to calculate the hydrolysis rate using the following equation:

$$ATP\;Hydrolysis\;Rate=\frac{\Delta OD/\min\;}{\Delta OD/\left[NADH\right]}\times \frac{1}{\left[Get3\;variant\right]}$$

## Data availability

The cryo-EM density map and atomic coordinates for the Get3_(D57N)_-Get4/5 structure reported in this publication have been deposited in the Protein Data Bank (PDB) and Electron Microscopy Data Bank (EMDB) under the following accession numbers: EMD-49743 and PDB 9NS5.

## Supporting information

This article contains supporting information. Additional data supporting this article, including supplementary figures (Figs. S1-S7) and a table (Table S1) are provided in the Supplementary Data document.

## Conflict of interest

The authors declare that they have no conflicts of interest with the contents of this article.
