# Supplementary material for: Get4/5-mediated remodeling of Get3's substrate-binding chamber: Insights into tail-anchored protein targeting by the GET pathway
Source: J Biol Chem. 2025 Sep 1;301(10):110667. doi: 10.1016/j.jbc.2025.110667 (PMC12506534; doi:10.1016/j.jbc.2025.110667)
Supplement: Supporting information [file mmc1.docx]

**Get4/5-Mediated Remodeling of Get3’s Substrate Binding Chamber:**

**Insights into Tail-Anchored Protein Targeting by the GET Pathway**

Diego Granados-Villanueva^1^, Andrew Rossow^2^, and Kelly H. Kim^1*^

^1^ Department of Biochemistry and Molecular Biology, Michigan State University, East Lansing, MI 48824, USA

^2^ Department of Biochemistry, University of Wisconsin-Madison, Madison, WI 53706, USA

*** Correspondence to:** [kimhyoj6@msu.edu](mailto:kimhyoj6@msu.edu)

**Running Title:** Get4/5-mediated Remodeling of Get3’s substrate binding chamber

**SUPPLEMENTARY DATA:**

**Supplementary Figure 1.** Assembly and purification of the Get3_(D57N)_-Get4/5 complex

**Supplementary Figure 2.** Cryo-EM data processing

**Supplementary Figure 3.** Cryo-EM data analysis

**Supplementary Figure 4.** Purification of Get3 variants used in mutagenesis studies

**Supplementary Figure 5.** MST analysis of Get3-Get4/5 interaction

**Supplementary Figure 6.** Confidence analysis of ColabFold-predicted structures for the

Get3-Get4/5 complex.

**Supplementary Figure 7.** Structural comparison of Yeast and Metazoan Get3-Get4/5
 Complexes.

**Supplementary Table 1.** Cryo-EM data collection and refinement statistics

**
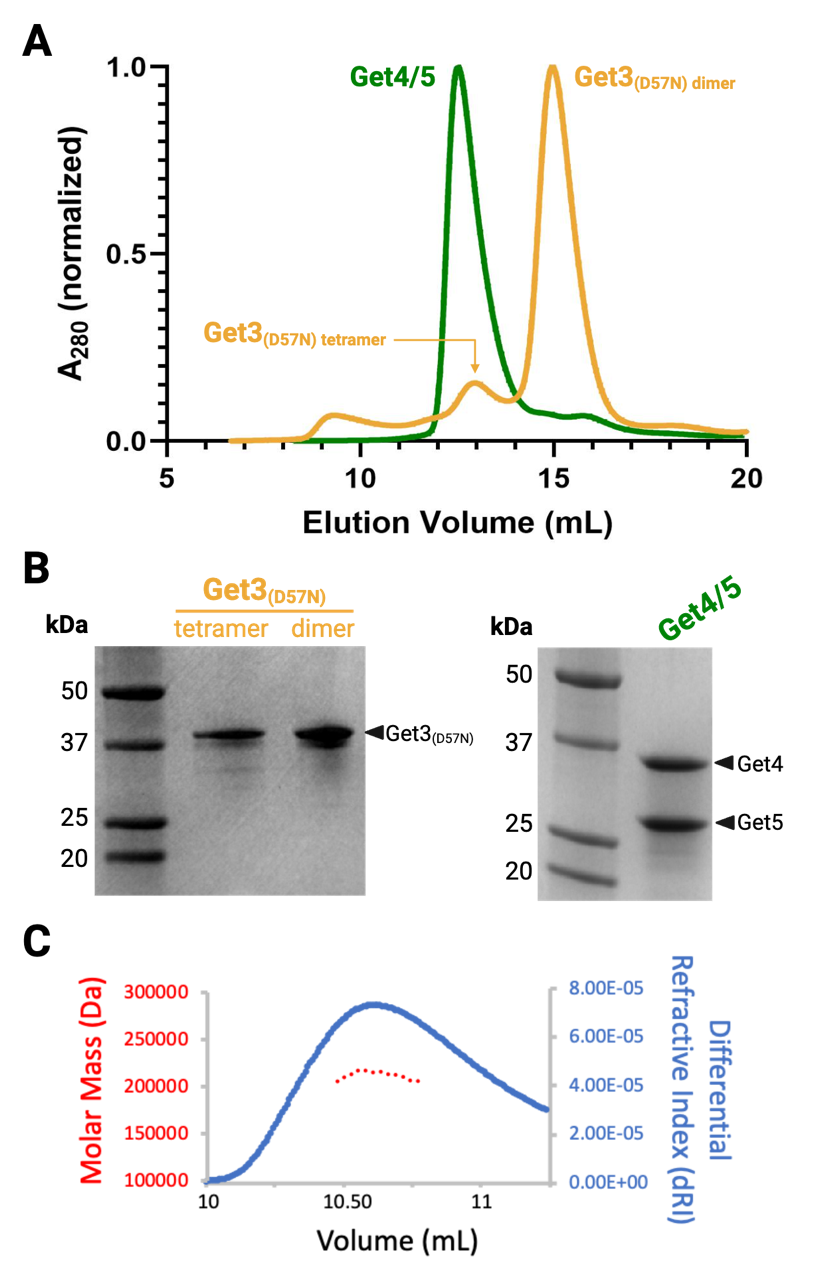
**

**Figure S1. Assembly and purification of the Get3_(D57N)_-Get4/5 complex. (A)** Size-exclusion chromatography (SEC) profiles of Get3_(D57N)_ (yellow) and Get4/5 (green). Get3_(D57N)_ eluted in two peaks corresponding to dimer and tetramer forms. The dimer fraction was used for reconstitution with Get4/5, which eluted as a heterotetramer. **(B)** SDS-PAGE of peak fractions from the SEC shown in (A). **(C)** SEC coupled with multiangle light scattering (SEC-MALS) analysis of the reconstituted Get3_(D57N)_-Get4/5 complex. The measured molecular mass is consistent with a complex containing one Get3 dimer and one Get4/5 heterotetramer.


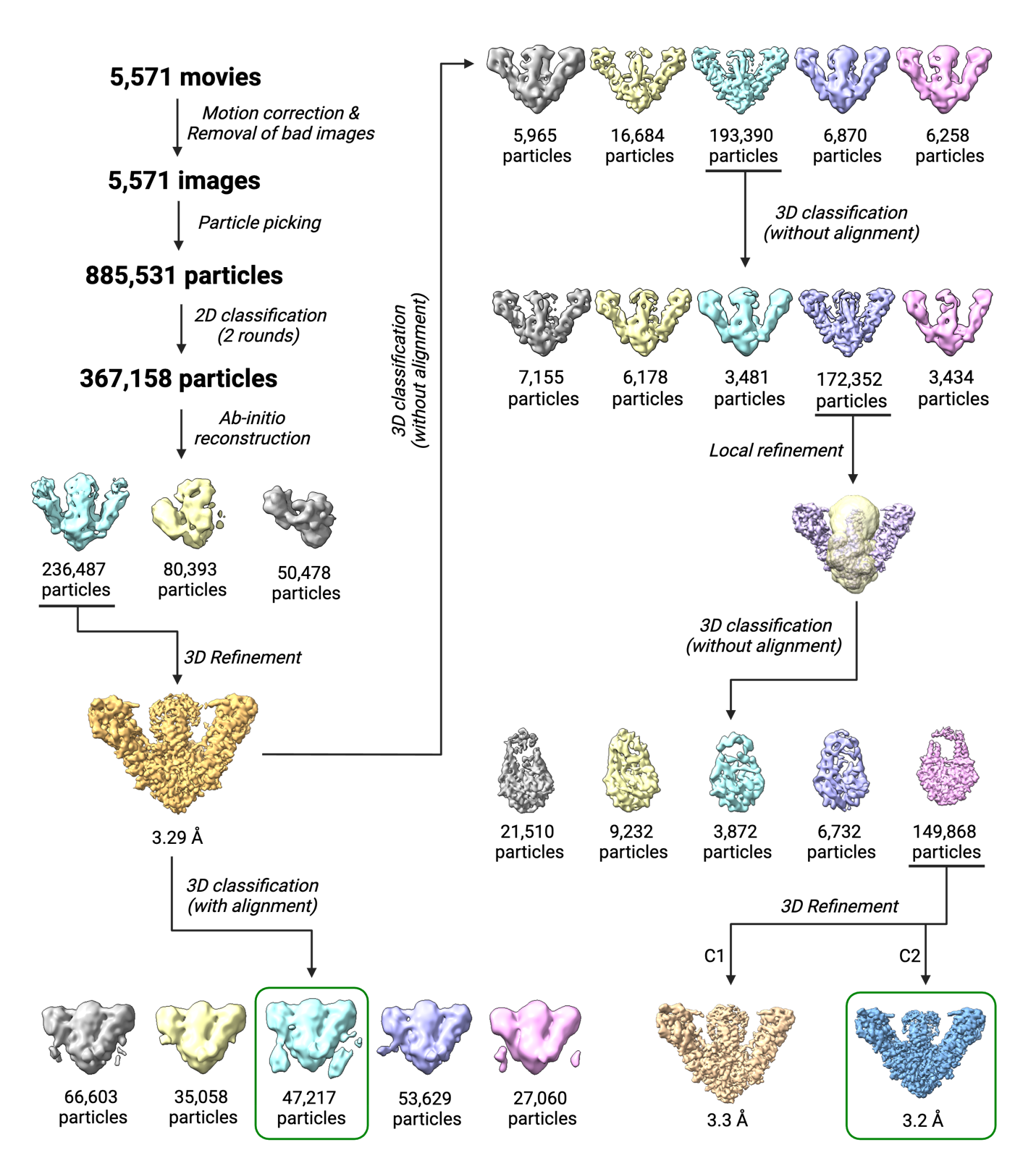


**Figure S2. Cryo-EM data processing.** Workflow used to determine the high-resolution cryo-EM structure of the Get3(D57N)-Get4/5 complex and to obtain maps showing density for the Sgt2-binding domain following 3D classification.

**
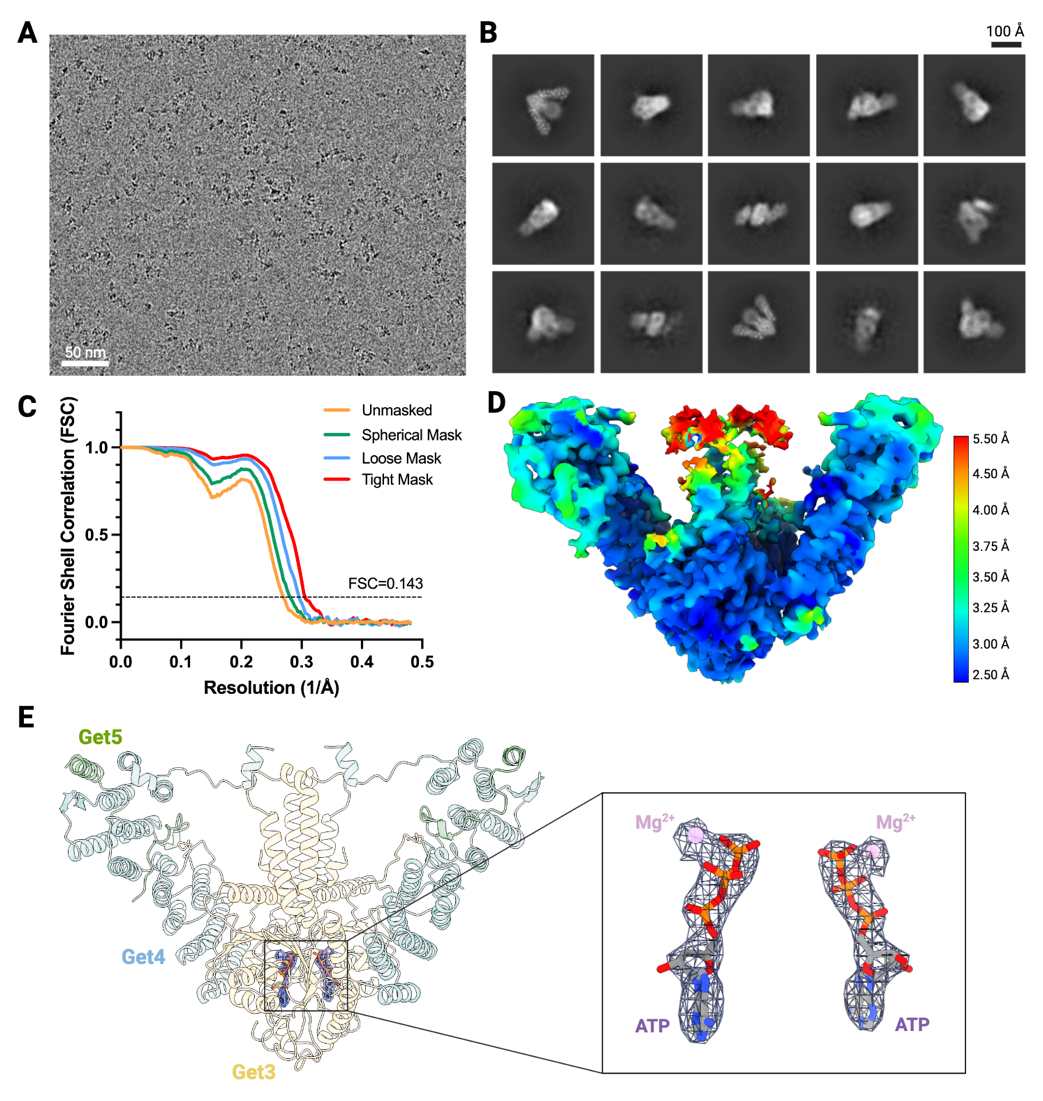
**

**Figure S3. Cryo-EM data analysis. (A)** Representative cryo-EM micrograph showing particles of the Get3_(D57N)_-Get4/5 complex embedded in vitreous ice. **(B)** Selected 2D class averages, revealing distinct orientations and structural features. **(C)** Fourier shell correlation (FSC) curves used to estimate the resolution of the final 3D reconstruction. FSC curves are shown for unmasked, spherical mask, loose mask, and tight mask conditions. The 0.143 cutoff criterion is indicated by the dashed line. **(D)** Final 3D cryo-EM reconstruction of the Get3_(D57N)_-Get4/5 complex colored by local resolution as estimated by CryoSPARC. The color scale bar indicates resolution in angstroms. **(E)** The locations of the two ATP molecules within the Get3_(D57N)_-Get4/5 complex are indicated, with the inset showing a close-up view of ATP and Mg^2+^ ions within well-resolved cryo-EM density map.

**
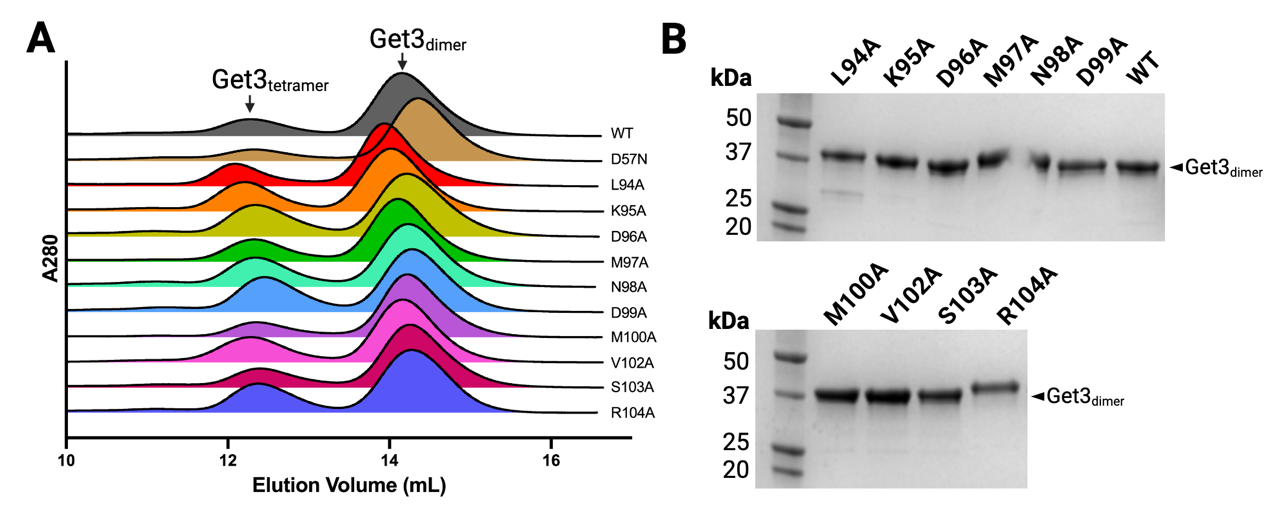
**

**Figure S4. Purification of Get3 variants used in mutagenesis studies. (A)** Size-exclusion chromatography (SEC) profiles of purified wild-type (WT) Get3 and single amino acid variants, including D57N, which lacks ATPase activity, as well as mutations located in the lateral gate region. All variants elute at positions corresponding to dimeric and tetrameric Get3. **(B)** SDS-PAGE analysis of peak fractions collected for the dimeric form of Get3, which is the physiologically relevant species and was used for structural and functional analyses in this study.

**
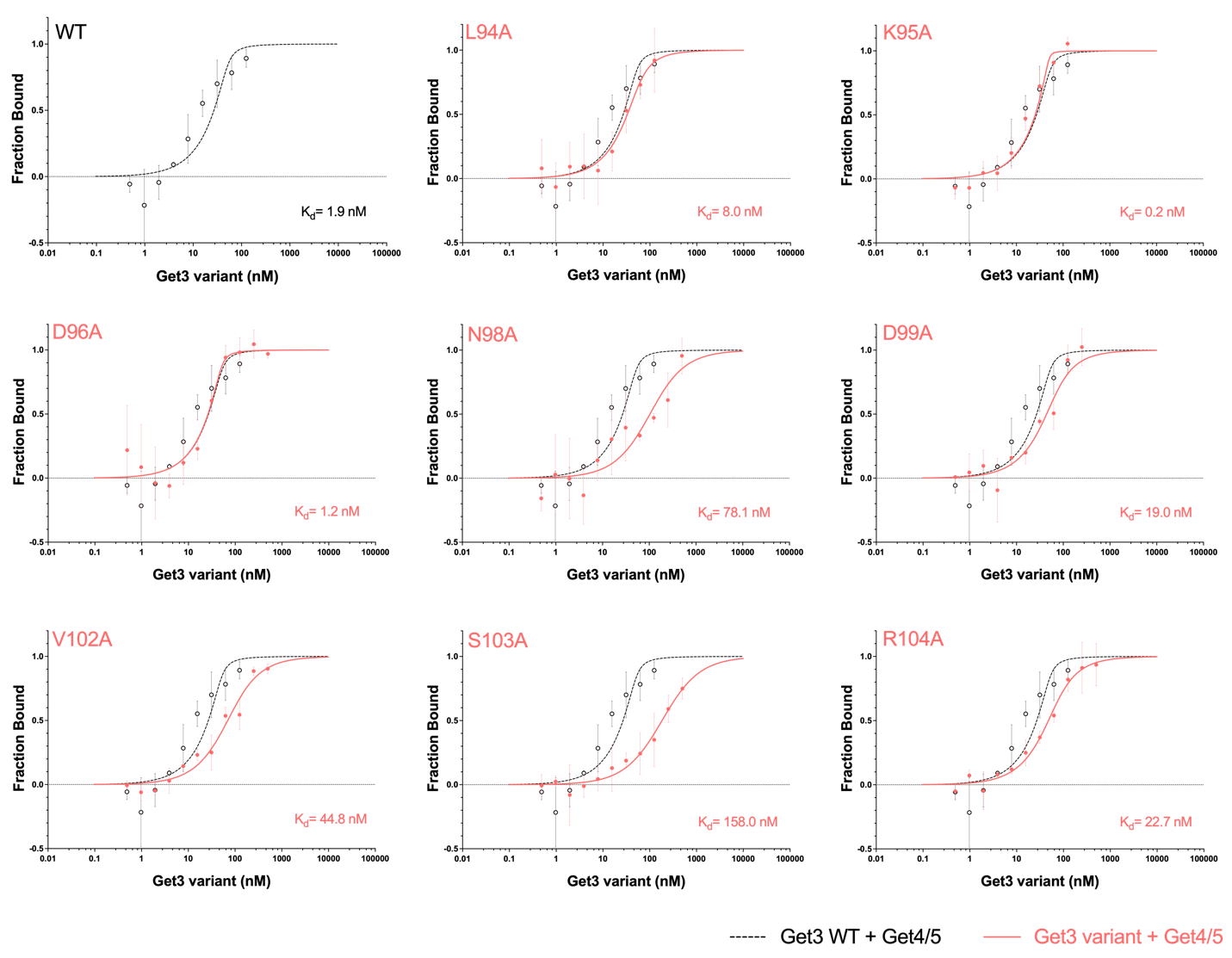
**

**Figure S5. Binding analysis of Get3 lateral gate variants to the Get4/5 by microscale thermophoresis (MST).** Equilibrium binding curves of purified Get3 wild-type (WT) and variants carrying single alanine substitutions in the lateral gate region (L94A, K95A, D96A, N98A, D99A, V102A, S103A, and R104A) measured by MST in the presence of Get4/5. Binding data were fit to a sigmoidal dose-response curve to determine apparent dissociation constants K_d_, with values indicated on each plot. Data points represent the mean ± SD from at least three independent experiments. The black dotted line shows the binding curve for Get3 WT with Get4/5, while the pink line represents the fitted binding curve for each Get3 variant with Get4/5.

**
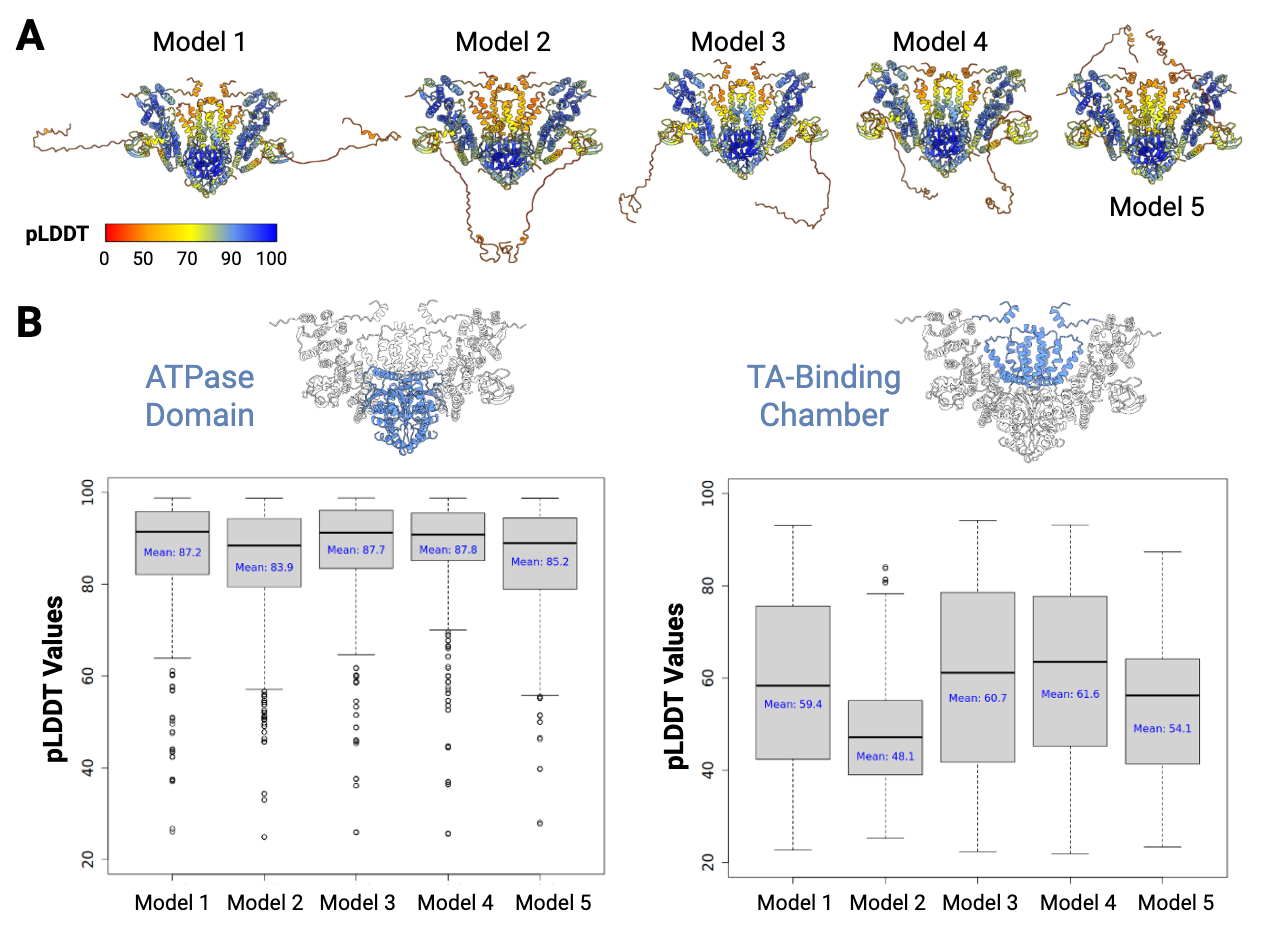
**

**Figure S6. Confidence analysis of ColabFold-predicted structures for the Get3-Get4/5 complex. (A)**Five structural models of the Get3-Get4/5 complex predicted by ColabFold, colored by per-residue pLDDT scores. Blue indicates high-confidence regions, while orange/red indicates low-confidence regions. **(B)**Box plots of pLDDT values for the ATPase domain (left) and TA-binding chamber (right) across all models. The analyzed region in each case is highlighted in blue on the structure of the Get3-Get4/5 complex shown above each plot. The ATPase domain displays consistently high confidence, whereas the TA-binding chamber shows lower and more variable confidence.

**
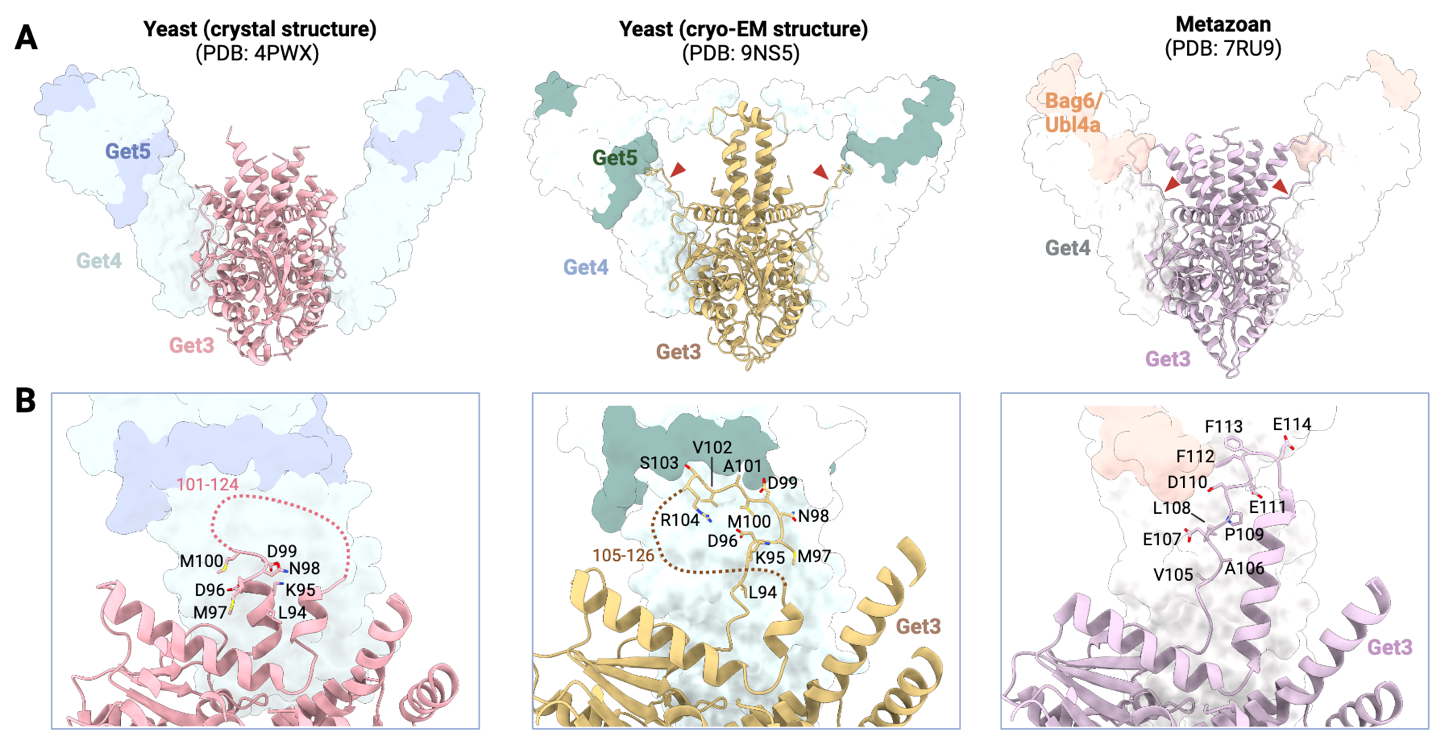
**

**Figure S7. Structural comparison of yeast and metazoan Get3-Get4/5 complexes. (A)**Crystal (left, PDB: 4PWX) and cryo-EM (middle, PDB: 9NS5) structures of the Get3-Get4/5 complex from yeast and metazoan (right, PDB: 7RU9). In the metazoan structure, portions of Ubl4a (Get5 ortholog) and Bag6 (an additional component in higher eukaryotes) have been removed for clarity. Red arrowheads indicate the position of the unfolded lateral gate region highlighted in panel B. **(B)**Close-up views of the lateral gate region unfolded upon interaction with Get4 in both complexes. The yeast Get3 structures (left and middle) are shown with key residues labeled and the missing residues represented by a dotted line. The corresponding metazoan Get3 structure is shown on the right.

**Table S1. Cryo-EM data collection and refinement statistics**

| ​ | **Get3(D57N)-Get4/5​**  EMD-49743  PDB 9NS5 |
| --- | --- |
|  |  |
| ***Data collection and processing*** |  |
| Magnification​ | 22,500 |
| Voltage (kV)​ | 300​ |
| Electron exposure (e/Å^2^)​ | 31.32​ |
| Defocus (μm)​ | -0.8 to -2.8​ |
| Pixel size (Å)​ | 1.0125 |
| Symmetry​ | C2​ |
| Initial particles​ | 885,531 |
| Final particles​ | 149,868 |
| Map resolution (Å)​ | 3.19​ |
| FSC threshold ​ | 0.143​ |
|  |  |
| ***Refinement*** |  |
| Model composition |  |
| Protein residues ​ |  |
| Get3_(D57N)_ | 326 |
| Get4 | 298 |
| Get5 | 48 |
| Non-hydrogen atoms | 10836 |
| Ligands | 3 |
| Average B-factor (Å^2^) ​ | ​159.436 |
| R.m.s. deviations ​(RMSD) | ​ |
| Bond lengths (Å) ​ | 0.003 |
| Bond angles (Å)​ | 0.552 |
| Validation |  |
| MolProbity score ​ | 1.72 |
| Clash score​ | 7.53 |
| Poor rotamers (%) | 0.66 |
| Ramachandran plot |  |
| Favored (%) | 95.63 |
| Allowed (%) | 4.22 |
| Outliers (%) | 0.15 |
